# A System of Practical Surgery

**Published:** 1904-04

**Authors:** 


A SYSTEM OF PRACTICAL SURGERY. By E. Van Bergmann, Von Bruns and Von Mikulicz. Edited and translated by W. T. Bull, M.D., and Walter Martin, M.D. Lea Bros. & Co., New York and Philadelphia, 1904.

This system is to consist of six volumes, and is to be sold by subscription. Each volume is of uniform size and fully illustrated. It is the work of those masters in surgery in Germany whose names are associated with the surgical art in its highest development. The American editor has not only superintended the translation, but has made such additions as appear necessary for the American reader. The work is a monumental one, and promises to supersede all others.

